# Supplementary material for: Mechanochemistry of phosphate esters confined between sliding iron surfaces
Source: Commun Chem. 2021 Dec 16;4:178. doi: 10.1038/s42004-021-00615-x (PMC9814736; doi:10.1038/s42004-021-00615-x)
Supplement: Supplementary file 1 — Supplementary Information [file 42004_2021_615_MOESM1_ESM.pdf]

Supplementary Information for: Mechanochemistry of Phosphate Esters Confined between Sliding Iron Surfaces

Carlos Ayestarán Latorre,[1,2] Joseph E. Remias,[3] Joshua D. Moore,[3,+] Hugh A. Spikes,[1] Daniele Dini,[1,4,5] James P. Ewen[1,4,5,*]

[1] – Department of Mechanical Engineering, Imperial College London, South Kensington Campus, London, SW7 2AZ, United Kingdom

[2] – Department of Materials, Imperial College London, South Kensington Campus, London, SW7 2AZ, United Kingdom

[3] – Afton Chemical Corporation, Richmond, Virginia 23219, United States of America

[4] – Institute of Molecular Science and Engineering, Imperial College London, South Kensington Campus, London, SW7 2AZ, United Kingdom

[5] – Thomas Young Centre for the Theory and Simulation of Materials, Imperial College London, South Kensington Campus, London, SW7 2AZ, United Kingdom

[+] – Current Address: Dassault Systèmes Americas Corporation, Waltham, Massachusetts 02451, United States of America

[*] – Corresponding author email: j.ewen@imperial.ac.uk

Supplementary Figures

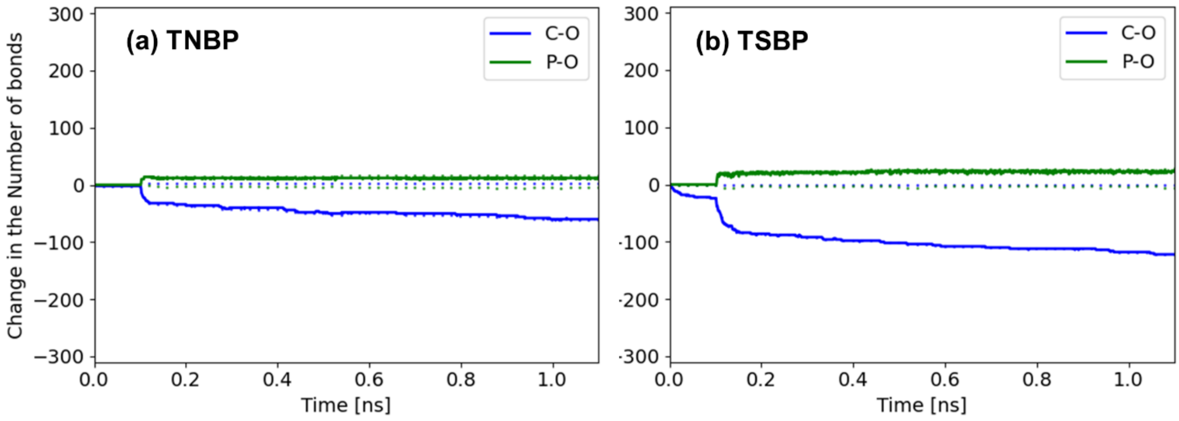

Supplementary Figure 1. Change in the number of intramolecular and intermolecular covalent bonds with sliding time for TNBP (a) and TSBP (b). Representative examples shown at 400 K and 2 GPa without sliding. Dashed lines show the individual contributions from bond formation (positive) and bond cleavage (negative), while dark lines show the change in the total number of bonds.

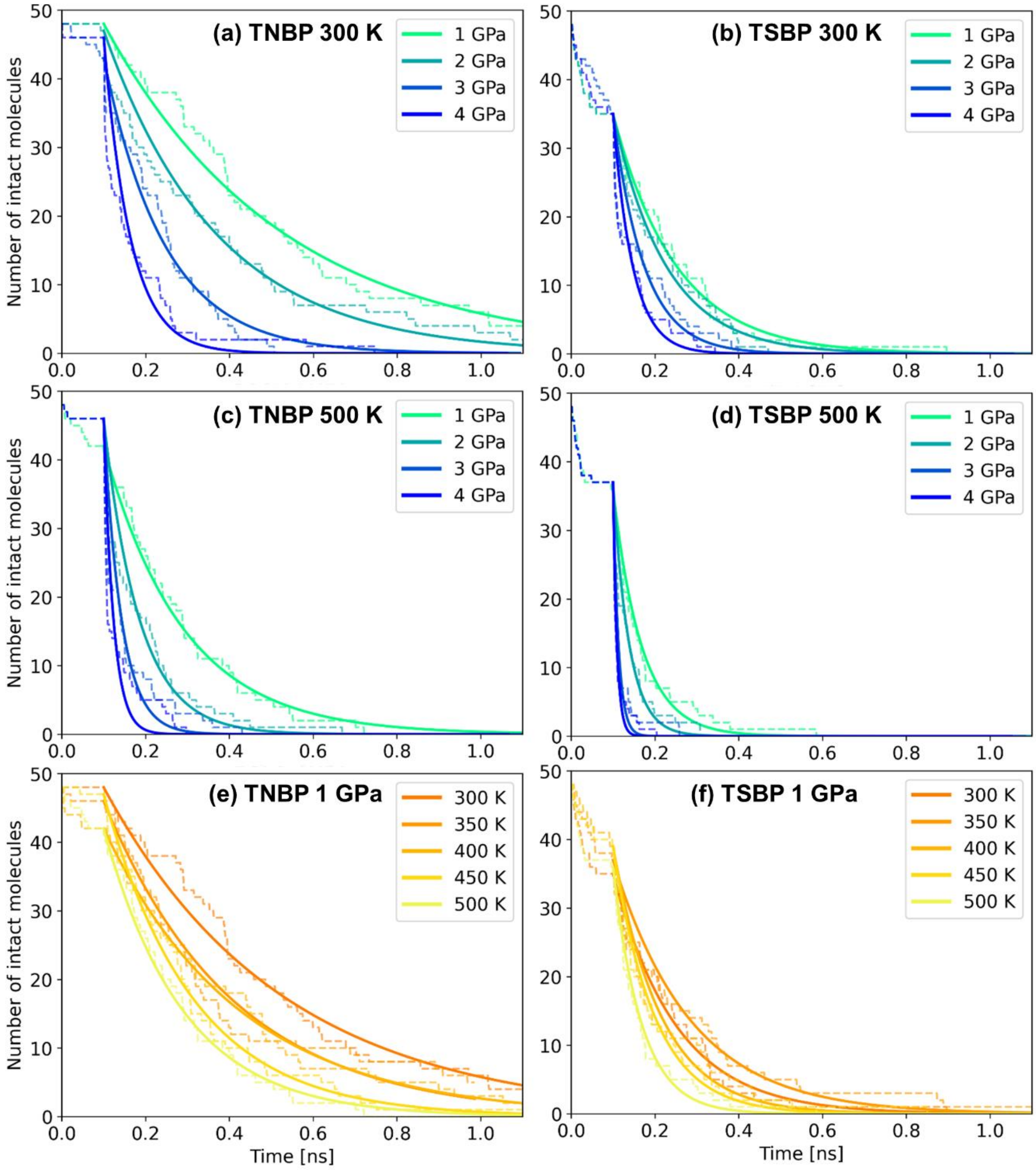

Supplementary Figure 2. Variation in the number of intact phosphate ester molecules with sliding time. Dashed lines are results from the NEMD simulations, solid lines are exponential decay fits to the data. The effect of pressure on the dissociation of TNBP (a) and TBSP (b) at low temperature (300 K). The influence of pressure on the dissociation of TNBP (c) and TBSP (d) at high temperature (300 K). The effect of temperature on the dissociation of TNBP (d) and TBSP (e) at low pressure (1 GPa).

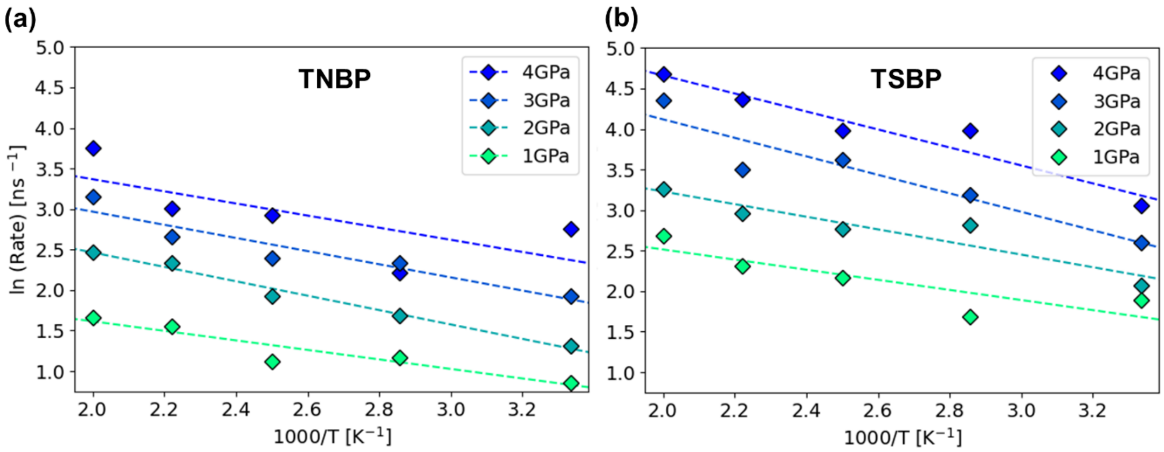

Supplementary Figure 3. Arrhenius plots of ln(rate) versus 1000/T for TNBP (a) and TSBP (b) obtained at different pressures. From Equation 1, the intercept of the dashed lines gives *A* and gradient gives E_a.

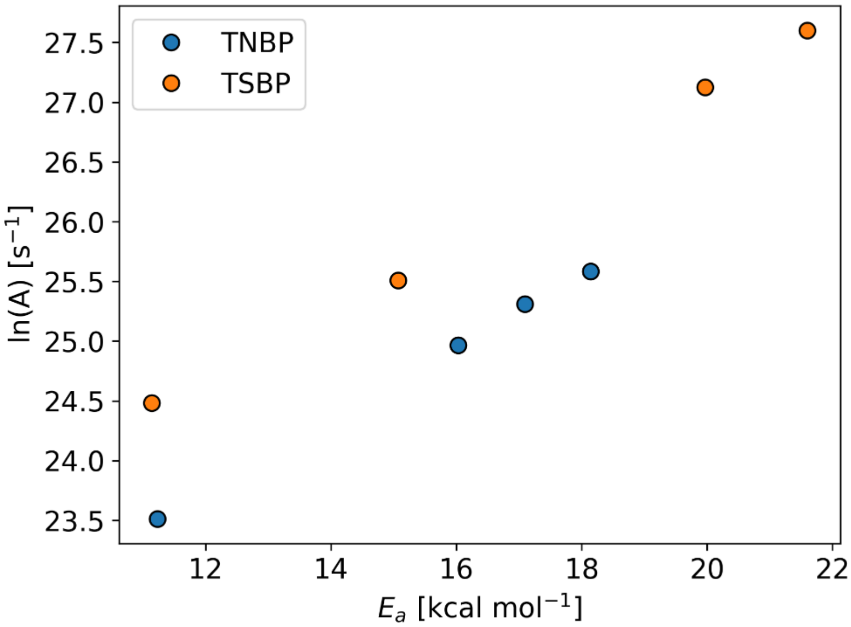

Supplementary Figure 4. Concurrent increase in E_a and ln(*A*) for TNBP and TSBP from the 2D fits showing a kinetic compensation effect.

Supplementary Table 1. Calculated values of the activation volume, ΔV^*, for TNBP and TSBP from the 2D fits to Equation 1. The parameter ranges represent the 95 % confidence intervals from the 2D fits.

	**ΔV^* [Å^3]**				
	**300 K**	**350 K**	**400 K**	**450 K**	**500 K**
**TNBP**	15 ± 8	10 ± 9	19 ± 11	17 ± 9	29 ± 4
**TSBP**	8 ± 3	24 ± 5	19 ± 19	27 ± 8	28 ± 24

Supplementary Table 2. Calculated values of the activation energy, E_a, and pre-exponential factor, A, for TNBP and TSBP from the 2D fits to Equation 1. The parameter ranges represent the 95 % confidence intervals from the 2D fits.

	**ln(A) [s^{-1}] ***				**E_a [kJ mol^{-1}] ***			
	**1 GPa**	**2 GPa**	**3 GPa**	**4 GPa**	**1 GPa**	**2 GPa**	**3 GPa**	**4 GPa**
**TNBP**	24 ± 1	25 ± 1	25 ± 1	26 ± 4	11 ± 4	16 ± 4	17 ± 5	18 ± 10
**TSBP**	25 ± 2	26 ± 1	27 ± 2	28 ± 2	11 ± 7	15 ± 7	20 ± 9	22 ± 9
* using mean ΔV^* from 300−500 K								

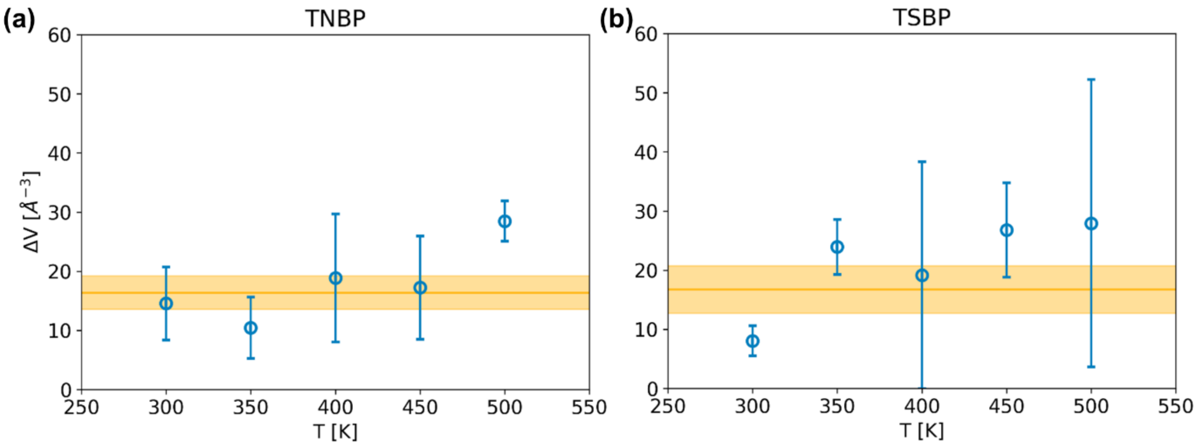

Supplementary Figure 5. Temperature dependence of ΔV^* for TNBP (a) and TSBP (b). Blue points are from the 2D fits, orange are from the 3D fits. Vertical bars indicate 95 % confidence intervals.

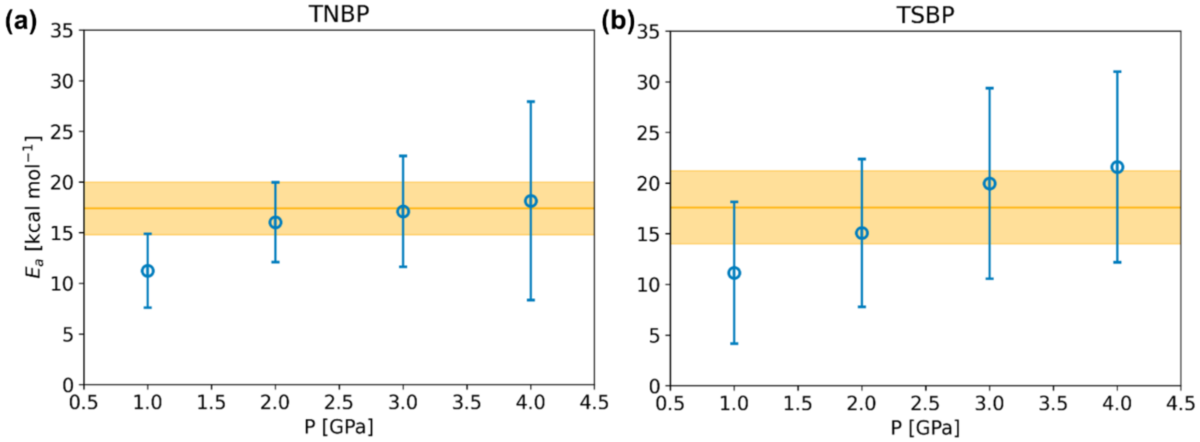

Supplementary Figure 6. Pressure dependence of E_a for TNBP (a) and TSBP (b). Blue points are from the 2D fits, orange are from the 3D fits. Vertical bars indicate 95 % confidence intervals.

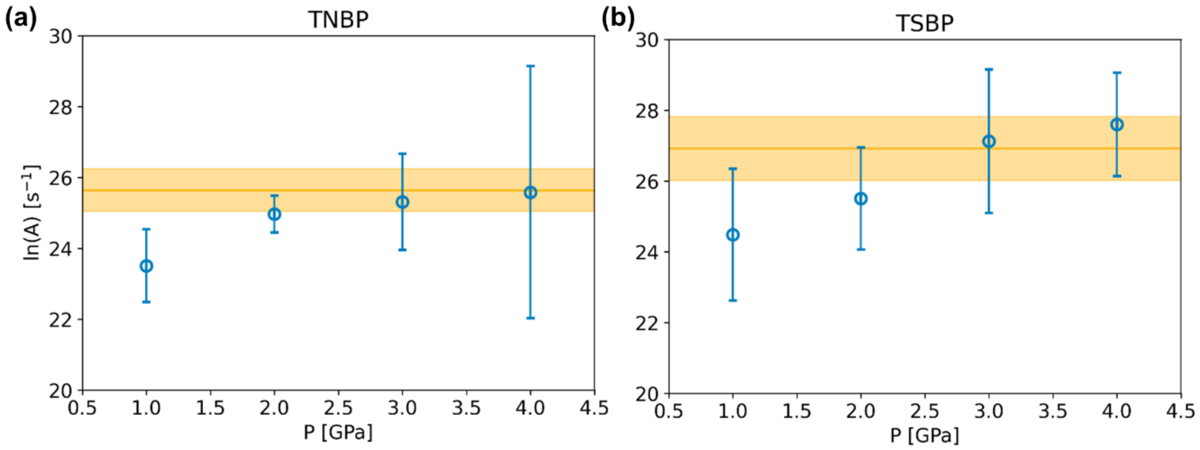

Supplementary Figure 7. Pressure dependence of ln(*A*) for TNBP (a) and TSBP (b). Blue points are from the 2D fits, orange are from the 3D fits. Vertical bars indicate 95 % confidence intervals.

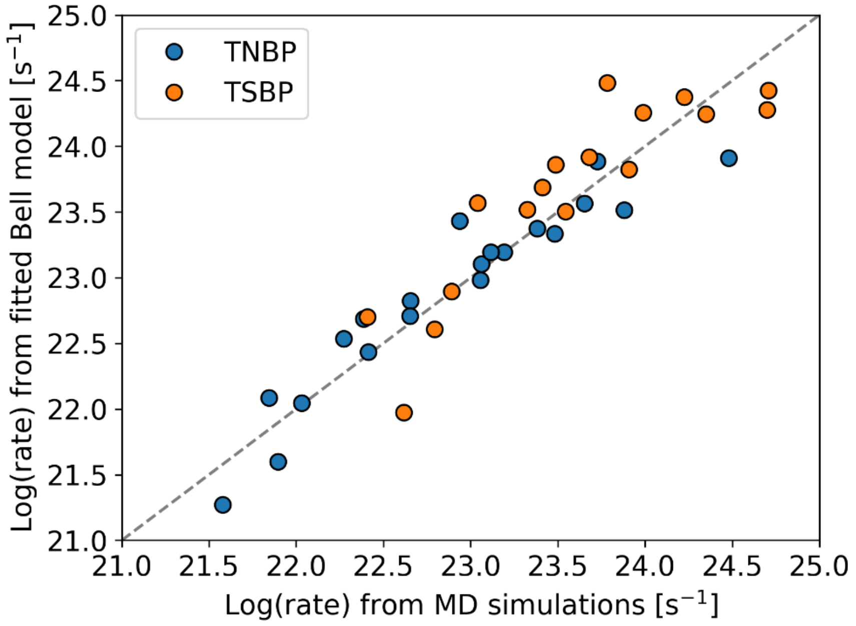

Supplementary Figure 8. Comparison of the logarithm of the reaction rates calculated from the NEMD simulations and those predicted using Equation 1. R^2 = 0.89 for TNBP and R^2 = 0.82 for TSBP.

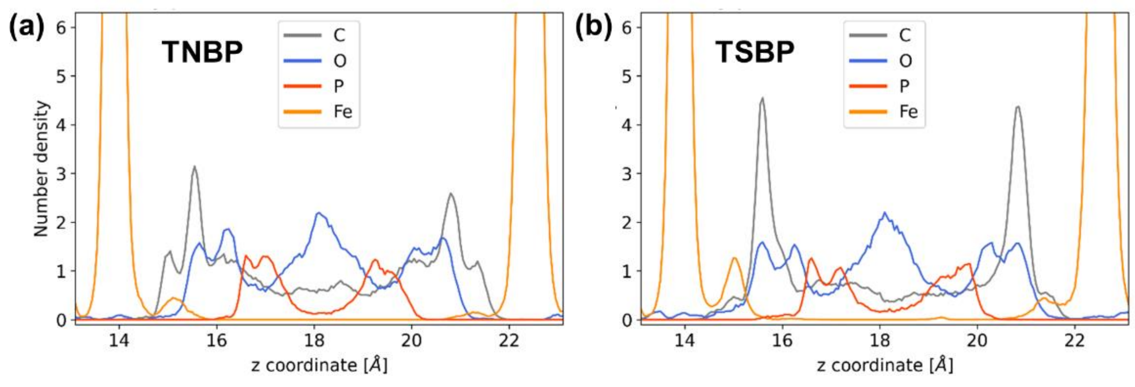

Supplementary Figure 9. Number density profiles for the C, O, and P atoms in the TNBP and TSBP molecules confined between the α-Fe(110) surfaces. Representative examples shown at 400 K and 2 GPa.

Supplementary Note

We estimate the confidence bounds, c, for the parameters used in the fits to Equation 1 by:

$$c = a \pm t_{n,\alpha/2} \sqrt{S} ,$$

where a are the coefficients obtained in the fits, $t_{n,\alpha/2}$ is the Student's *t* score for *n* degrees of freedom and $1 - \alpha$ confidence level, and S are the diagonal elements from the estimated covariance matrix of the coefficients.

We performed equal variance t-tests to check if the values for the reaction constants for TNBP and TSBP from the 3D fits can be determined to be different. For the 38 degrees of freedom of our samples and an alpha level of 5 %, the t-value for rejection is 2.024. For the kinetic parameters, the calculated t-value from the equal variance t-tests is:

	t-value
$\ln(A)$	114.5
E_a	2.8×10^{-8}
ΔV^*	1.7×10^{-7}

This means we can confidently reject the hypothesis of pre-exponential factors being equal for TNBP and TSBP, while the values for E_a and ΔV^* are essentially identical.
