# Supplementary material for: Mechanochemistry of phosphate esters confined between sliding iron surfaces
Source: Commun Chem. 2021 Dec 16;4:178. doi: 10.1038/s42004-021-00615-x (PMC9814736; doi:10.1038/s42004-021-00615-x)
Supplement: Supplementary file 2 — Description of Additional Supplementary Files [file 42004_2021_615_MOESM2_ESM.pdf]

Description of Additional Supplementary Files

File Name: Supplementary Data

Description: Data used to produce plots in Figures 1-4 in the main text.
